# Oat-Protein-Based Diet Lowers Blood Pressure and Prevents Cardiac Remodeling and Dysfunction in Spontaneously Hypertensive Rats

**DOI:** 10.3390/nu16223870

**Published:** 2024-11-13

**Authors:** Pema Raj, Jenny Bouchard, Delphine Martineau-Côté, Lovemore Malunga, Lamia L’Hocine, Liping Yu, Babak Sobhi, Allaoua Achouri, Mélanie Pitre, Sijo Joseph Thandapilly, Thomas Netticadan

**Affiliations:** 1 St. Boniface Hospital Research Centre, Winnipeg, MB R2H 2A6, Canada; 2 Richardson Center for Food Technology and Research, Winnipeg, MB R3T 2N2, Canada; 3 Department of Human Food and Human Nutritional Sciences, University of Manitoba, Winnipeg, MB R3T 2N2, Canada; 4 Agriculture and Agri-Food Canada, Saint-Hyacinthe Research and Development Centre, Saint-Hyacinthe, QC J2S 8E3, Canada; 5 Agriculture and Agri-Food Canada, Winnipeg, ON K1A 0C5, Canada; 6 Canadian Centre for Agri-Food Research in Health and Medicine, Winnipeg, MB R2H 2A6, Canada; 7 Department of Physiology and Pathophysiology, University of Manitoba, Winnipeg, MB R3T 2N2, Canada

**Keywords:** oat protein, hypertension, cardiac remodeling, cardiac dysfunction, inflammation, antioxidant, nitric oxide

## Abstract

**Background/Objectives:** Hypertension and its associated complications, such as cardiac remodeling and dysfunction, continue to impose a significant burden on global healthcare. Nutritional interventions have been recognized as playing a crucial role in addressing this devastating condition termed a ‘silent killer’. Plant-based proteins could potentially be utilized as a non-pharmacological strategy to combat hypertension and its related risk factors. In this study, we investigated the efficacy of an oat protein diet in managing hypertension and cardiac abnormalities. **Methods:** Four-week-old male spontaneously hypertensive rats (SHRs) and Wistar–Kyoto (WKY) rats were fed a regular diet with casein as a protein source or an oat-protein-based diet for 16 weeks. Twenty-week-old male SHRs showed high blood pressure (BP), cardiac remodeling, cardiac dysfunction, higher levels of markers of oxidative stress [malondialdehyde (MDA)] and inflammation [tumor necrosis factor-α (TNF-α)], as well as lower levels of a marker of vascular function (nitric oxide). **Results:** The oat protein diet was able to significantly lower high BP, prevent cardiac remodeling and dysfunction, improve the levels of nitric oxide, and reduce the levels of TNF-α. Oat protein, after *in vitro* gastrointestinal digestion, also exhibited angiotensin-converting enzyme inhibition and significantly higher antioxidant activity than casein when assessed with the 2,2-diphenyl-1-picrylhydrazyl and the iron-chelating assays *in vitro.* **Conclusions:** oat protein lowers BP and prevents cardiac remodeling and dysfunction partly via improving the levels of nitric oxide and TNF-αin SHRs. Its high antioxidant potential could contribute to the observed cardiovascular effects.

## 1. Introduction

Hypertension is a debilitating disease significantly impacting approximately 1.28 billion patients and thereby contributing to a large number of premature deaths in the global population [1]. Alarmingly, approximately 75% of individuals with hypertension do not have their condition under control with medical management [1]. Blood pressure regulation is carried out involving many organ systems. The renin–angiotensin–aldosterone system (RAAS) is crucial in systemic blood pressure regulation. The renin produced by the kidneys converts angiotensinogen to angiotensin I (in active form), and angiotensin-converting enzyme (ACE) cleaves it to produce the active form, angiotensin II (Ang II). Ang II causes vasoconstriction by acting on venous and arterial smooth muscle. Dysregulation of RAAS leads to excessive amounts Ang II, causing hypertension. ACE also breaks down bradykinin, which possesses vasodilatory properties. Ang II, in turn, causes the increase in vasopressin production and aldosterone secretion, which lead to sodium and water retention and potassium elimination [2]. Both these processes contribute to increased BP. Oxidative stress involves the formation of highly reactive peroxynitrate radicals when superoxide radicals interact with nitric oxide (NO), significantly reducing NO bioavailability. Reactive oxygen species (ROS) is known to uncouple endothelial nitric oxide synthase (eNOS) from L-arginine oxidation, generating more ROS instead of NO, resulting in endothelial dysfunction and impairing endothelium-dependent relaxations, which largely contribute to hypertension development [3]. Uncontrolled hypertension significantly contributes to the development of congestive heart failure, end-stage renal disease, and stroke. Various interconnected factors are reported to play a role in the onset of hypertension, including genetic predisposition, heightened sympathetic nervous system activity, increased peripheral vascular resistance, endothelial dysfunction, impaired NO synthesis, elevated oxidative stress, and imbalance in the RAAS [4]. 

Along with guideline-directed medical therapy, a major breakthrough in hypertension management via dietary approaches has been attained with the Dietary Approaches to Stop Hypertension (DASH) diet, comprising fruits, vegetables, whole grains, nuts, seeds, and legumes [5,6]. Moreover, the Coronary Artery Risk Development in Young Adults (CARDIA) also revealed an inverse relationship between plant-based food consumption and blood pressure (BP) in a dose dependent manner [7]. Similarly, The International Study of Macro- and Micro-Nutrients (INTERMAP) showed that vegetable protein intake was inversely related to BP [8]. In the Chicago Western Electric Study, increased consumption of plant proteins was linked to lower BP changes observed over an 8-year follow-up period [9]. Soy protein, pea protein, rice protein, and their peptide have been shown to possess antihypertensive action due to their ACE inhibitory action [10]. On account of these promising avenues, further research is necessary to fully understand the effectiveness of plant-based proteins as a strategy for managing hypertension and enhancing cardiovascular health.

Oat is a whole grain known for its well-proportioned nutritional composition, including its high fiber content, which offers substantial health benefits [11]. Oat protein has been increasingly considered as an environmentally friendly plant protein source that has better sensory characteristics that can be incorporated into a balanced diet [11]. Oat protein is an excellent source of all essential amino acids, apart from lysine [12,13]. Oats and oat protein are naturally low in saturated fat, which is beneficial for cardiac health [12,14]. Moreover, oat protein holds promise as a significant player in the alternative dairy and meat market, emerging as a novel protein ingredient [12]. The oat protein market is reported to have grown from USD 48 million in 2018 to an estimated USD 63 million by 2025, with a 4.1% annual growth rate from 2019 to 2025 [15]. Importantly, oat protein is currently underexplored as a plant-protein-based nutritional strategy to manage hypertension and other chronic diseases and may hold promise due to its unique characteristics.

The BP-regulating activities of oats were reviewed earlier by our group [3]. However, the antihypertensive effects are due to its dietary fiber, β-glucan. The protein fraction in oats has not been fully explored with respect to its capacity to ameliorate hypertension. Preliminary *in vitro* and *in silico* studies have identified a variety of bioactive peptides derived from oat protein that may possess ACE-inhibiting, renin-inhibiting, antioxidant, immunomodulating, α-amylase-inhibiting, and DPP-IV-inhibiting activities, suggesting potential cardioprotective and antidiabetic effects [12,16,17]. Emerging evidence indicates that oat protein affords protection from hypercholesterolemia and hyperglycemia and an antioxidant advantage. Oat protein is known to reduce total cholesterol and LDL-C by regulating the activities of liver CYP7A1 and HMG CoAR, rate-limiting enzymes in the bile acid and cholesterol synthesis pathways, in high-fat- and high-fat-, high-sugar-fed animal models [2,18,19]. Furthermore, a recent study from our group demonstrated that oat protein was effective in preventing systolic dysfunction in a diet-induced metabolic syndrome model [2]. Based on these results, oat protein may be an effective dietary agent to reduce cardiovascular disease risk factors.

Several animal models of hypertension have been utilized to elucidate pathophysiology and therapeutic interventions. Spontaneously hypertensive rat (SHR), the Dahl salt-sensitive rat, the Lyon hypertensive rat, the Sabra hypertensive rat, and the inherited-stress-induced arterial hypertension model are rodent models of human hypertension. The SHR model is the most commonly studied model for essential human hypertension. SHRs are well recognized for their predisposition to develop early primary hypertension and display notable cardiac abnormalities similar to those found in humans with hypertension. In SHRs, hypertension develops around 5–6 weeks of age. In the adult phase, SHRs develop many characteristics of cardiovascular disease such systolic blood pressure reaching 180–200 mmHg, increased oxidative stress, cardiac hypertrophy, vascular dysfunction, as well as systolic and diastolic dysfunction, eventually progressing to heart failure at 18–24 months [4]. Therefore, the SHR model has been one of the best available animal models for exploring antihypertensive potential therapies. In the current study, we hypothesized that oat protein would be beneficial in controlling BP and alleviating cardiac abnormalities in the chronic hypertension setting. Therefore, we investigated the potential of an oat protein diet to lower BP and prevent cardiac remodeling and dysfunction in male SHRs in this diet-feeding study.

## 2. Materials and Methods

### 2.1. Animal Ethics

The animal experimental protocols for this project were approved by the University of Manitoba Office of Research Ethics and Compliance and Animal Care Committee and were conducted in accordance with the guidelines by the Canadian Council for Animal Care ((AC11568) Protocol # 20-021/1, 8 December 2021).

### 2.2. Oat Protein Extraction and Oat-Protein-Feeding Study Design

Manitoba-sourced oats, provided by Buffalo Creek Mills Altona, MB, Canada, were used for producing all the oat protein needed for this study. Oat protein was prepared using a wet extraction method as described by us in an earlier study [20]. Briefly, the protein was extracted from defatted oat flour with alkaline water (pH 9.5) at 35 °C for 1 h. The mixture was centrifuged at 5000× *g* for 15 min, and the protein was then collected from the supernatant through isoelectric point (pH 4.5–5.5) precipitation. The protein pellet was mixed with water, and its pH was adjusted to 7.0–7.5 with 4 N NaOH. The protein extract was freeze-dried and milled prior to its incorporation into a rat chow provided by Research Diets Inc., (New Brunswick, NJ, USA). Male SHRs served as the model of human primary hypertension in this study. Male Wistar–Kyoto (WKY) rats were used as normotensive controls. The cardiac hypertrophy seen in SHRs is similar to the development of hypertrophy due to hypertension in humans [21,22]. The SHR strain was produced by the selective inbreeding of normal WKY rats with hypertension, which is why WKY rats were used as controls in our study. In SHRs, hypertension is present from 6 weeks of age. At 2 months of age, SHRs exhibit heart dysfunction, and heart failure is seen around 18 months of age [21,22]. Four-week-old SHRs and WKY rats were obtained from Charles River Inc. (Montreal, QC, Canada). SHRs and WKY rats were assigned to 3 different experimental groups (Schema 1) and fed 20–25 g of either an AIN93G-based control diet (one group of WKY rats and one group of SHRs) with casein as the protein source (n = 10) or an AIN93G diet including oat protein (protein purity: 88.9%) (n = 10) daily (one group of SHRs) for a period of 16 weeks based on the standard caloric and nutritional needs (Research Diets Inc., New Brunswick, NJ, USA). Food intake was monitored daily. At 20 weeks of age, this study was terminated, and the heart tissue and blood were collected. The tissue was stored at −80 °C after flash freezing with liquid nitrogen.

### 2.3. Transthoracic Echocardiography

Cardiac structure and function were assessed in all three experimental groups with transthoracic echocardiography after 16 weeks of feeding. The rats were anesthetized during the procedure (isoflurane 3%). Transthoracic two-dimensional (2D)-guided M-mode and pulse-wave Doppler measurements were performed using an ultrasound system (VIVID E9, GE, USA) with a 13 MHz transducer, as previously described by us [16,17]. The parameters measured included the left ventricular ejection fraction (LVEF), fractional shortening (FS), cardiac output (CO), interventricular septal wall thickness IVS), left ventricular posterior wall thickness at end (LVPW), left ventricular internal dimensions (LVID), and isovolumetric relaxation time (IVRT). Echo cine images were obtained from 3 cardiac cycles and analyzed using EchoPAC software (GE, Boston, MA, USA).

### 2.4. BP Measurements

BP measurements were performed on conscious animals after 16 weeks of treatment. Systolic and diastolic BP measurements were carried out by using a tail-cuff sphygmomanometer (Kent Scientific, Torrington, CT, USA), as described by us previously [16]. Throughout each measurement cycle, a volume–pressure-recording cuff pushed blood away from the tail. The back flow of the blood was stopped using an occlusion cuff. Once the occlusion cuff deflated, the blood flow was let back into the tail, which increased the tail volume. Systolic BP was measured as the pressure exerted by the occlusion cuff during the increase in tail volume. Diastolic BP was measured as the occlusion cuff pressure during the deflation at which the blood flow in the tail equalized.

### 2.5. Biochemical Assessments

Serum lipid peroxidation levels were estimated using an assay kit to determine the amount of malondialdehyde (MDA), a lipid peroxidation product (Abcam, Waltham, MA, USA), as described previously [23]. The serum concentrations of the inflammatory cytokine tumor necrosis factor-α (TNF-α) was determined using an ELISA assay kit (Abcam, Waltham, MA, USA). The serum concentrations of norepinephrine, angiotensin II, and nitric oxide were determined using assay kits as described previously [23]. Using a Cobas c111 clinical chemistry analyzer (Roche Diagnostics, Laval, QC, Canada), the plasma concentrations of alanine aminotransferase (ALT), aspartate aminotransferase (AST), urea, glucose, creatinine, total cholesterol (TC), high-density lipoprotein cholesterol (HDL-C), low-density lipoprotein cholesterol (LDL-C), and triglycerides (TGs) were measured as described previously [20].

### 2.6. In Vitro Antioxidant and ACE Inhibition Activities of Oat Protein in Comparison to Casein After In Vitro Gastrointestinal Digestion

#### 2.6.1. *In Vitro* Gastrointestinal Digestion Protocol

Oat- and casein-based diets, oat proteins, and casein were digested *in vitro* according to the INFOGEST harmonized static *in vitro* gastrointestinal digestion protocol [24]. Briefly, 3 g of ground diet was mixed in a ratio 1:1 with simulated salivary fluid (SSF) containing α-amylase from the porcine pancreas (75 U/mL digestate), and digestion was conducted for 2 min at 37 °C under constant stirring. Casein and oat protein isolates were diluted in water prior to the oral phase to reach a similar protein content to the oat- and casein-based diets (0.6 g of protein and 2.4 g of water). For the gastric phase, the digestate was mixed in a 1:1 ratio with simulated gastric fluid (SGF)-containing pepsin (2000 U/mL digestate). The digestion was carried out at 37 °C and pH 3.0 for 2 h under constant stirring. The digestates were diluted once more for the intestinal digestion phase, in a ratio 1:1 with the simulated intestinal fluid (SIF)-containing pancreatin from the porcine mucosa (100 U trypsin activity/mL digestate) and 10 mM bile salts; the pH was adjusted to 7.0. The digestates were then incubated for 2 h at 37 °C under constant stirring. At the end of the intestinal phase, digestates were cooled on ice, and 5 mM 4-(2-aminoethyl) benzenesulfonyl fluoride hydrochloride (AEBSF) was added to inhibit enzymes. The digestates were centrifuged at 15,000× *g* for 30 min at 4 °C, and the supernatants were filtered using an Amicon ultrafiltration system equipped with a 3 kDa molecular weight cut-off (MWCO)-regenerated cellulose membrane (EMD Millipore, Billerica, MA, USA) to recover small-molecular-weight peptides. The collected 3 kDa permeates were aliquoted and frozen at −80 °C for further analysis.

#### 2.6.2. Assessment of Protein Degree of Hydrolysis

The degree of protein hydrolysis (DH) was determined to compare the digestibility extent of the diet and their protein components. The method was performed following the procedure of Adler-Nissen [2] and Spellman et al. [25], with minor modifications. Briefly, the digestate supernatants were appropriately diluted in 1% SDS (*m*/*v*). L-leucine standards at different concentrations (32–262 mg/L) were prepared in the same solvent. Volumes of 0.25 mL of the standard and diluted samples were added to test tubes and mixed with 2 mL of 0.2125 M phosphate buffer at pH 8.2 and 2 mL of 0.1% (*m*/*v*) 2,4,6-trinitrobenzenesulfonic acid (TNBS) reagent (diluted in water). The tubes were incubated for 1 h at 50 °C in a water bath. After the incubation period, 4 mL of 0.1 N HCL was added to stop the reaction. The absorbance was recorded at λ = 340 nm, and the primary amino group contents in the samples are expressed as mg of leucine equivalent. Those values were used to calculate the degree of hydrolysis (DH), which was defined as the percentage of peptide bonds cleaved during the *in vitro* digestion procedure using the following formula:

$$DH\;\left(\%\right)=\frac{{(AN}_{2}-{AN}_{1})}{N_{p}}\times 100$$

where AN_2_ and AN_1_ are the primary amino group contents expressed as mg of leucine equivalent normalized to the protein content in the digestate (after enzyme contribution subtraction) and in the undigested sample (no enzyme), respectively. N_p_ is the primary amino group content of totally hydrolyzed samples subjected to acidic hydrolysis conditions (6 N HCl) and incubated at 110 °C for 24 h. Enzyme contribution was assessed by performing digestion with water instead of samples.

#### 2.6.3. *In Vitro* ACE Inhibition Activity

The potential ACE inhibition activity of the oat protein and casein gastrointestinal digestate permeates was evaluated *in vitro* according to Barbana and Boye [26], as described in Martineau-Côté et al. [27]. Briefly, various concentrations of 3 kDa permeate of oat and casein digestates were mixed with ACE from rabbit lung (Sigma-Aldrich, St. Louis, MO, USA) in a test tube and equilibrated at 37 °C for 10 min. Then, 50 μL of the substrate was added (1 mM hippury-L-histidyl-L-leucine (HHL)) and the reaction was carried out for 30 min at 37 °C. We added 85 μL of HCL 1N par to stop the reaction, and the samples were analyzed by HPLC using a 4.60 × 250 mm Aqua C18 column (5 µm pore size 125 Å; Phenomenex, Torrance, CA, USA). The samples were eluted in isocratic mode with 50% (*v*/*v*) methanol in water containing 0.1% trifluoroacetic acid (*v*/*v*) at a flow rate of 0.5 mL/min for 15 min, and elution was monitored at 228 nm. The peak area of HHL was recorded, and ACE inhibition was calculated as follows:

$$ACE\;Inhibition\;\left(\%\right)=\left(1-\frac{\left({PA}_{a}-{PA}_{b}\right)}{\left({PA}_{a}-{PA}_{c}\right)}\right)\times 100$$

where PA_a_ is the peak area of the control (HHL without ACE and samples; PA_b_ is the peak area of the samples (HHL, ACE, and inhibitory peptides), and PA_c_ is the peak area of the reaction blank (HHL and ACE, without samples). The IC_50_ was calculated using a nonlinear regression with a four-parameter logistic curve of the ACE inhibition (%) plotted against its respective peptide concentration. The IC_50_ was defined as the peptide concentration required to inhibit 50% of ACE activity.

#### 2.6.4. *In Vitro* Antioxidant Activity

Different *in vitro* assays were used in combination to target various antioxidant mechanisms of action. The 2,2-diphenyl-1-picrylhydrazyl (DPPH), the 2,2 ′-azinobis(3-ethylbenzothiazoline-6-sulfonic acid) (ABTS), the oxygen radical absorption capacity (ORAC), and the iron-chelating assays were performed following the methods of Orona-Tamayo et al. [28], Re et al. [29] and Tomer et al. [30] respectively, as described in Martineau-Côté, Achouri, Wanasundara, Karboune, and L’Hocine [27]. For the DPPH, ABTS and iron-chelating assays, various concentrations of the 3 kDa permeate of oat and casein digestate were tested to evaluate the dose-response effect. The results are reported as the half-maximal effective concentration (EC_50_), which was defined as the peptide concentration leading to a 50% scavenging or chelating activity. The EC_50_ values were calculated using nonlinear regression with a four-parameter logistic curve of the scavenging or chelating activity plotted against the peptide concentration. For the ORAC assay, the results are expressed as µmol of Trolox equivalent per mg of peptides.

#### 2.6.5. *In Silico* Prediction of Antihypertensive, Antioxidant, and Anti-Inflammatory Activities of Casein and Oat Proteins After Gastrointestinal Digestion

An *in silico* analysis was also performed to assess the potential antihypertensive and antioxidant activities of casein and oat proteins after gastrointestinal digestion. The main oat storage proteins (Prolamin, 11S and 12S globulins) and bovine caseins sequences were retrieved from the UniProtKB database [31]. The protein accession numbers were as follows: β-casein (P02666), α-s1 casein (P02662), α-s2 casein (P02663), κ-casein (P02668), 12S globulin (P12615), 11S globulin (Q38780), and Avenin-9 (Q09114). The protein sequences were digested *in silico* with pepsin (pH> 2) (EC 3.4.23.1), trypsin (EC 3.4.21.4), and αchymotrypsin (EC 3.4.21.1) using the Bioactive Peptide Database of University of Warmia and Mazury (BIOPEP-UWM) enzyme tool [32]. The obtained peptide profile was screened for ACE inhibitor, renin inhibitor, as well as antioxidant and anti-inflammatory peptide fragments. The frequency of fragments with such activities (A_E_) were calculated by the BIOPEP-UWM algorithm.

### 2.7. Statistical Analysis

All values are expressed as means ± SEM. One-way ANOVA was used to compare the differences between the experimental groups using GraphPad Prism 5 software. Tukey’s post hoc test was used to further evaluate the statistical significance when a simple effect was detected between groups. A *p* value of <0.05 was statistically significant. Each analysis for *in vitro* activity was performed in triplicate, and the results are expressed as mean ± standard deviation (SD). The data were analyzed through two-way analysis of variance (ANOVA) (*p* < 0.05) followed by Tukey’s honest significant difference (HSD) post hoc test (*p* < 0.05) using he XLSTAT software (https://www.xlstat.com/en/; Addinsoft, New York, NY, USA).

## 3. Results

**Figure 1:**
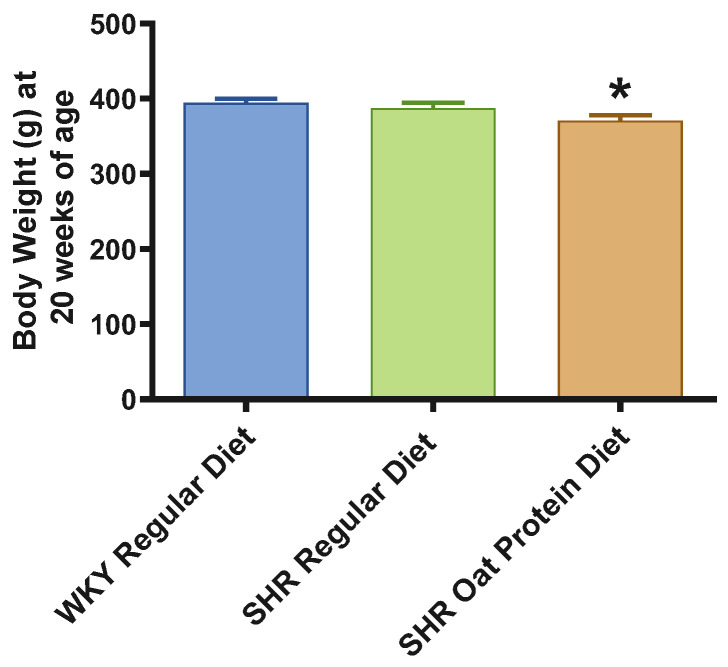
Effects of oat protein on body weight of 20-week-old male WKY rats and SHRs. Values are means ± SEMs (n = 10 rats). Significant values are defined as *p* < 0.05. * *p* < 0.05 vs. WKY regular diet.

**Figure 2:**
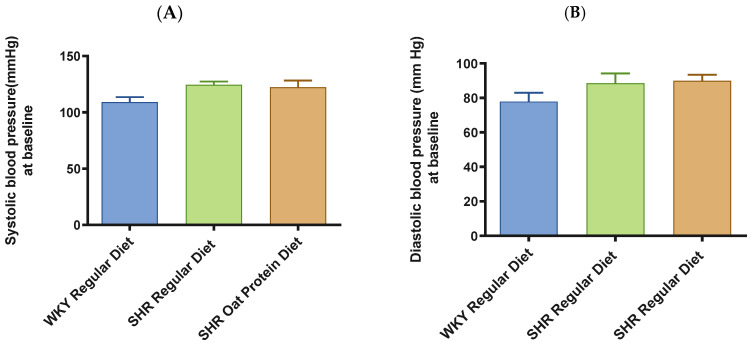
Effects of oat protein on BP at baseline in 5-week-old male WKY rats and SHRs. (**A**) Systolic BP in male WKY rats and SHRs at baseline, (**B**) systolic BP in female WKY rats and SHRs at baseline. Values are means ± SEMs (n = 10 rats).

**Figure 3:**
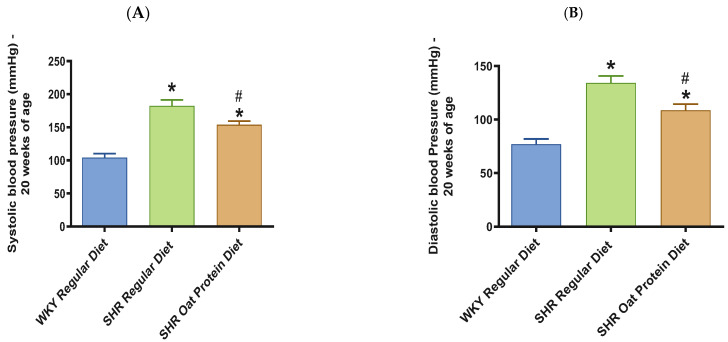
Effects of oat protein on BP in 20-week-old WKY rats and SHRs. (**A**) Systolic BP in male WKY rats and SHRs; (**B**) diastolic BP in male WKY rats and SHRs. Values are means ± SEMs (n = 9 rats). Significant values are defined as *p* < 0.05. * *p* < 0.05 vs. WKY regular diet. # *p* < 0.05 vs. SHR regular diet.

SHRs fed an oat protein diet had significantly lower body weight compared to SHRs fed a regular diet (Figure 1). BP was obtained before starting the treatment at baseline (0 weeks—all animals were 4 weeks of age at baseline BP measurements) and at the end of this study after 16 weeks of treatment (all animals were 20 weeks of age) to determine the progressive changes in the BP of the SHRs compared to the WKY rats and to delineate the efficacy the oat protein diet in managing hypertension. Systolic and diastolic BPs were comparable, without any significant statistical difference at baseline (Figure 2A,B). Systolic and diastolic BPs were significantly elevated in 20-week-old male SHRs that received a casein-based regular diet when compared to male WKY rats receiving a casein-based regular diet (Figure 3A,B). In contrast, the systolic and diastolic BPs were significantly lower in 20-week-old male SHRs that received the oat protein diet than in male SHRs that received the casein-based regular diet (Figure 3A,B).

**Figure 4:**
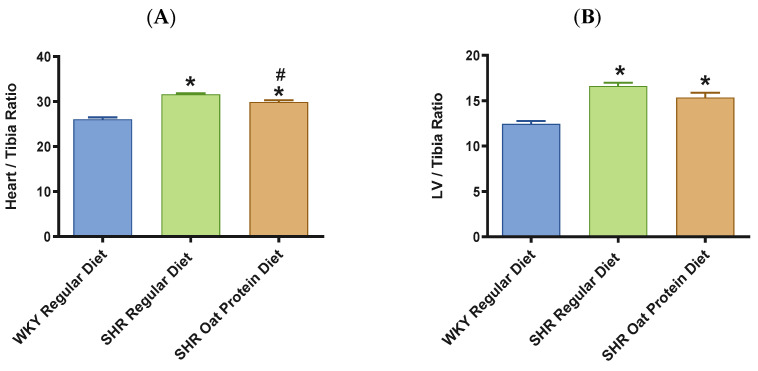
Effects of oat protein on heart/tibia (**A**) and LV-to-tibia-length ratio (**B**) after 15 weeks of treatment: male WKY rats and SHRs. Values are means ± SEMs (n = 10 rats). * *p* < 0.05 vs. WKY regular diet. # *p* < 0.05 vs. SHR regular diet.

**Figure 5:**
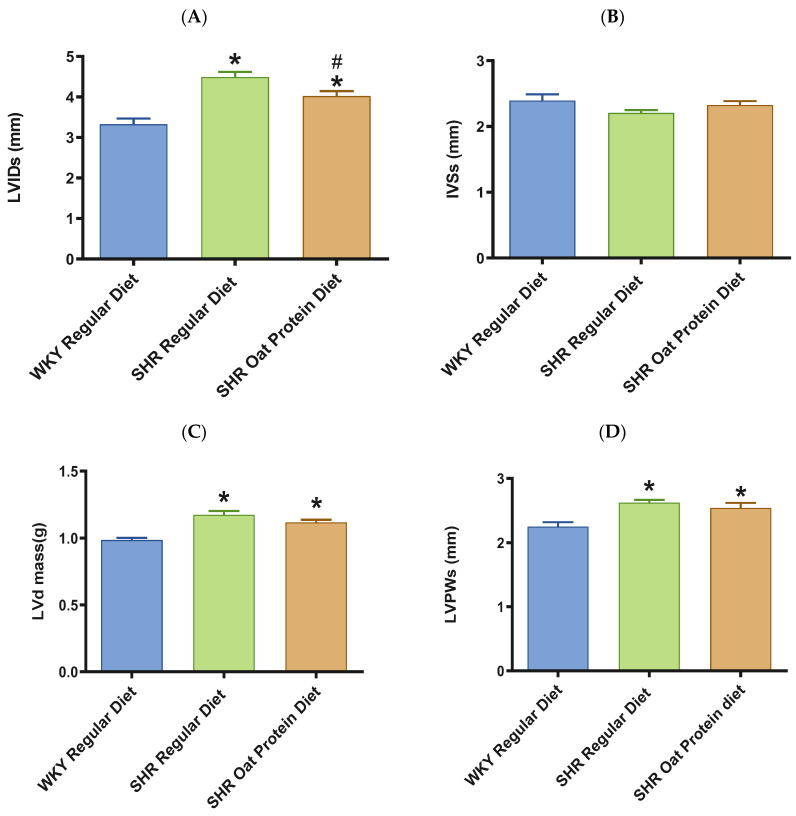
Effects of oat protein on cardiac structure of 20-week-old male WKY rats and SHRs (after 15 weeks of treatment). (**A**) Left ventricular internal dimension (LVID) at systole in male WKY rats and SHRs. (**B**) Interventricular septal wall thickness (IVS) at systole in male WKY rats and SHRs. (**C**,**D**) Left ventricular mass (LVd mass), left ventricular posterior wall thickness (LVPW) at systole in male WKY rats and SHRs. Values are means ± SEMs (n = 10 rats). Significant values are defined as *p* < 0.05. # *p* < 0.05 vs. WKY + W, * *p* < 0.05 vs. SHR + W.

The heart/tibia length ratio was measured as a surrogate marker of cardiac hypertrophy. The heart/tibia length and LV-to-tibia ratio were higher in male SHRs consuming a regular diet when compared to age-matched male WKY rats (Figure 4A,B). Male SHRs fed the oat protein diet had a lower heart/tibia length ratio than the age-matched male SHRs on a regular diet without affecting the LV-to-tibia ratio. Transthoracic echocardiography analysis showed that 20-week-old male SHRs fed a regular diet had increased internal dimensions of the heart chamber, such as the left ventricle (LVID), when compared to age-matched male WKY rats fed the regular diet (Figure 5A). The LVID was smaller in male SHRs on the oat protein diet compared to male SHRs on the regular diet. The other cardiac chamber structural parameters such as cardiac wall thickness and IVS were not altered between the groups at 20 weeks of age (Figure 5B). However, the LVPW and LV mass were found to be significantly higher in male SHRs on both the regular diet and oat protein diet than in WKY rats, despite a trend evidently indicating a shift toward positive remodeling (Figure 5C,D).

**Figure 6:**
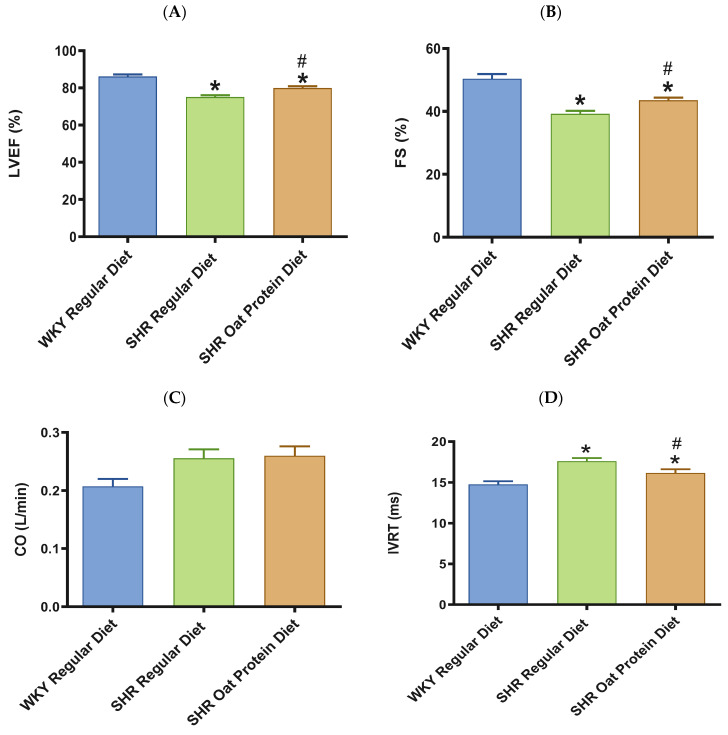
Effects of oat protein on cardiac function of 20-week-old male WKY rats and SHRs after 15 weeks of treatment. (**A**) Left ventricular ejection fraction (LVEF), (**B**) fractional shortening (FS), (**C**) cardiac output (CO), (**D**) isovolumic relaxation time (IVRT). Values are means ± SEMs (n = 10 rats). Significant values are defined as * p < 0.05 vs. WKY regular diet. # *p* < 0.05 vs. SHR regular diet.

In terms of cardiac systolic function, there was a significant reduction in the systolic functional parameters LVEF and FS in 20-week-old male SHRs fed a regular diet in comparison to male WKY rats receiving a regular diet (Figure 6A,B). SHRs fed an oat protein diet were found to have significantly higher LVEF and FS values when compared to SHRs fed a regular diet (Figure 6A,B). In our current study, CO was statistically no different between the groups (Figure 6C). In this study, there was a significant prolongation in the IVRT in 20-week-old male SHRs on a regular diet in comparison to male WKY rats on a regular diet (Figure 6D). Oat protein resulted in a reduction in the IVRT in male SHRs, suggesting that this treatment was effective in preventing diastolic dysfunction by improving LV relaxation.

**Figure 7:**
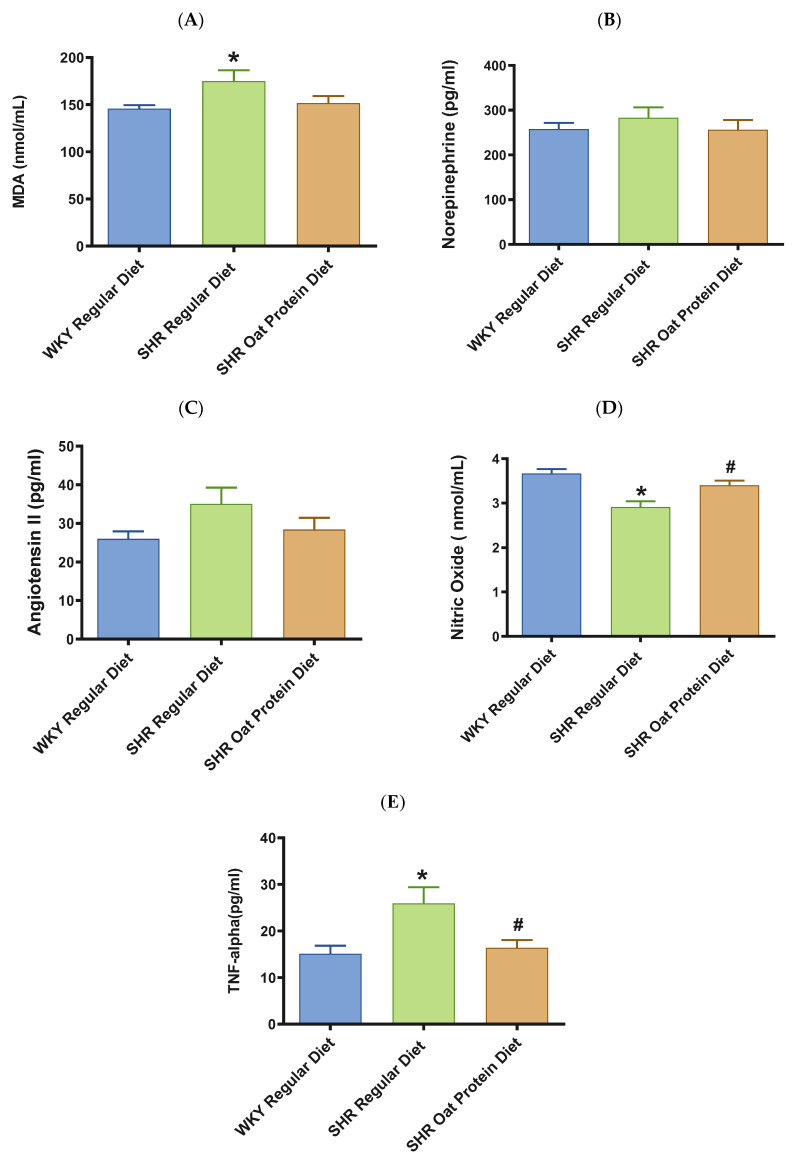
Effects of beta-glucan on (**A**) serum malondialdehyde (MDA), (**B**) norepinephrine, (**C**) angiotensin II, (**D**) nitric oxide, and (**E**) tumor necrosis factor-α (TNF-α) in 20-week-old male WKY rats and SHRs. Values are means ± SEMs (n = 10 rats). Significant values are defined as *p* < 0.05. * *p* < 0.05 vs. WKY regular diet. # *p* < 0.05 vs. SHR regular diet.

Male SHRs on the regular diet had significantly higher serum MDA (a marker of oxidative stress) in comparison to male WKY rats on the regular diet at 20 weeks (Figure 7A). The serum MDA levels in the male SHRs on an oat protein diet were not significantly different when compared to those of the male SHRs on a regular diet. The levels of vasoconstrictor molecules norepinephrine and Ang II were comparable in 20-week-old rats between the groups (Figure 7B,C). The serum levels of nitric oxide (measured as nitrate/nitrite) were significantly lower in male SHRs on a regular diet than on WKY rats on a regular diet. The serum levels of nitric oxide were significantly higher in male SHRs on the oat protein diet than in SHRs on the regular diet (Figure 7D).

TNF-α was significantly higher in male SHRs on the regular diet in comparison to male WKY rats on the regular diet at 20 weeks (Figure 7E). TNF-alpha was significantly lower in male SHRs on the oat protein diet in comparison to male SHR rats on the regular diet at 20 weeks.

**Figure 8:**
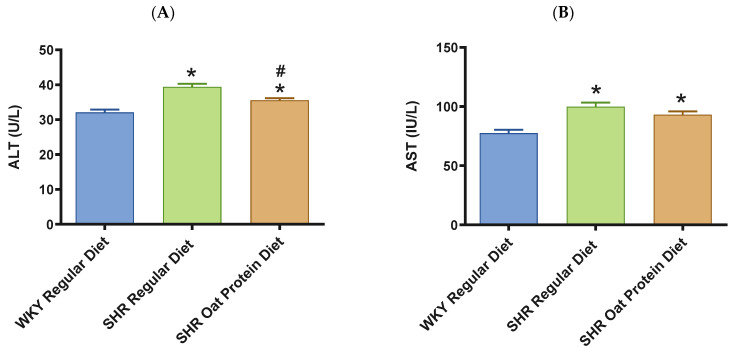

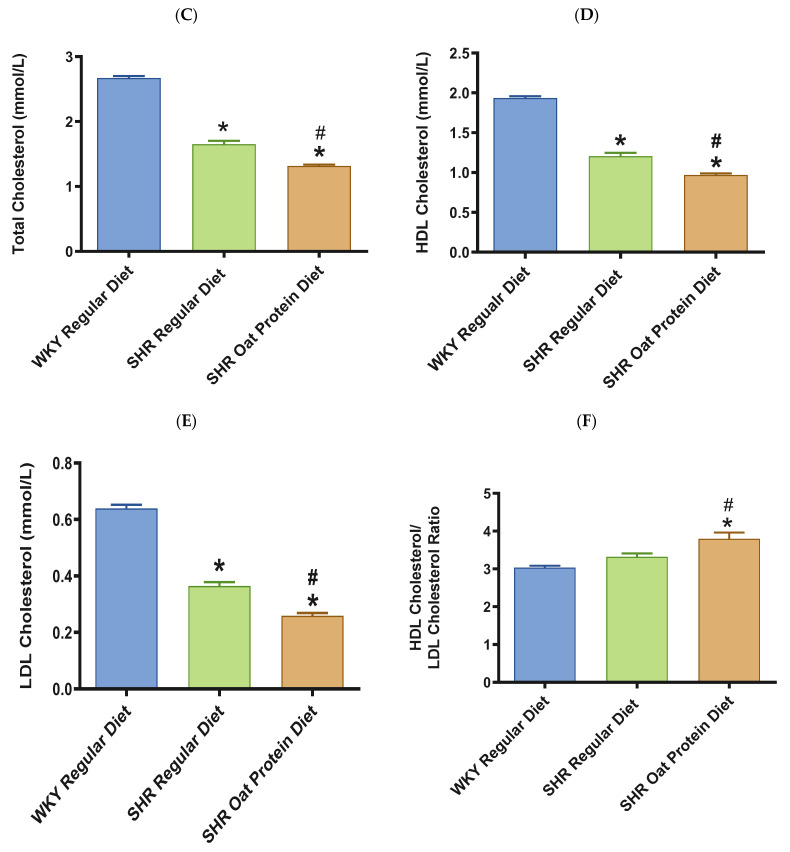

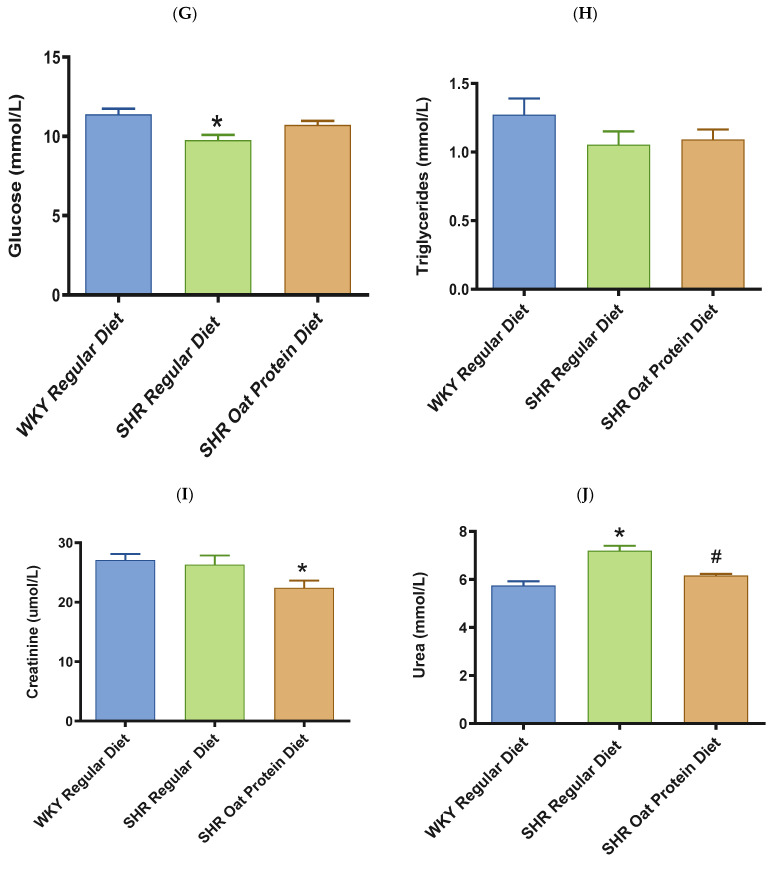
The effects of oat protein on serum alanine aminotransferase (ALT) (**A**), aspartate aminotransferase (AST) (**B**), total cholesterol (**C**), high-density lipoprotein cholesterol (HDL-C) (**D**), low-density lipoprotein cholesterol (LDL-C) (**E**), HDL-C/LDL-C (**F**), glucose (**G**), triglycerides (**H**), creatinine (**I**), and urea (**J**) after 16 weeks of treatment. Values are means ± SEMs. Significant values are defined as * *p* < 0.05. vs. WKY regular diet. # *p* < 0.05 vs. SHR regular diet.

ALT and AST were significantly higher in male SHRs fed a regular diet in comparison to male WKY rats fed a regular diet at 20 weeks (Figure 8A,B). ALT but not AST was significantly lower in male SHRs on the oat protein diet than in male SHRs on the regular diet at 20 weeks. Total cholesterol was significantly lower in male SHRs on a regular diet than in WKY rats on aregular diet (Figure 8C). Total cholesterol was also significantly lower in male SHRs on the oat protein diet than in SHRs and WKY rats on the regular diet. HDL cholesterol was significantly lower in male SHRs on s regular diet compared to WKY rats on s regular diet as well (Figure 8D). Male SHRs fed oat protein also had significantly lower HDL cholesterol than male SHRs on a regular diet. Similarly, LDL cholesterol was significantly lower in male SHRs on a regular diet than in WKY rats on a regular diet (Figure 8E). LDL cholesterol was significantly lower in male SHRs fed an oat protein diet than in SHRs fed a regular diet. Male SHRs fed oat protein also had a significantly higher HDL cholesterol/LDL cholesterol ratio compared to male WKY rats and SHRs on a regular diet (Figure 8F). Triglycerides were comparable between the groups, whereas glucose levels were lower in SHRs fed a regular diet (Figure 8G,H). Creatinine levels were comparable between the male SHRs and WKY rats on the regular diet (Figure 8I). Creatinine levels were significantly lower in male SHRs on the oat protein diet. The urea level was significantly higher in male SHRs on a regular diet compared to WKY rats on a regular diet (Figure 8J). Additionally, the urea level was significantly lower in male SHRs on the oat protein diet compared to male SHRs on the regular diet.

To gain an insight on the potential mechanism of action explaining the BP-lowering effect and the cardioprotective effects of oat proteins when fed to SHR rats, casein- and-oat protein-based feeds, as well as isolated casein and oat proteins, were digested *in vitro*, and the bioactive potency of the digestates was assessed. The degree of protein hydrolysis was determined, as well, to evaluate their digestibility extent.

**Figure 9:**
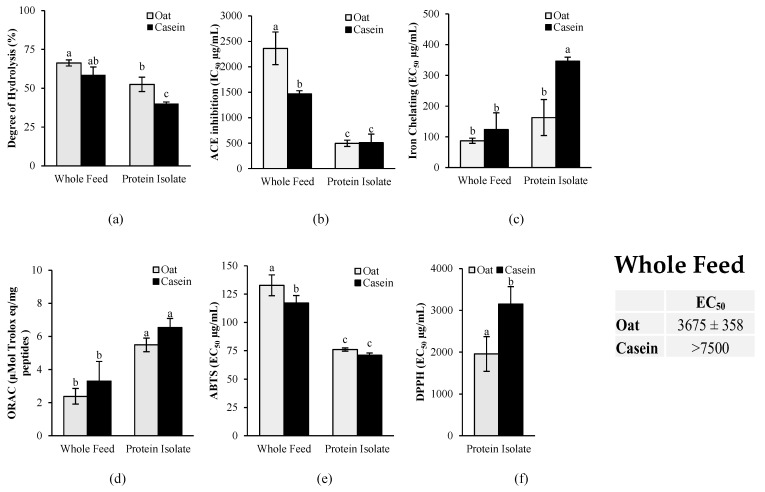
Antihypertensive and antioxidant activities of casein and oat proteins after *in vitro* gastrointestinal digestion: (**a**) degree of protein hydrolysis; (**b**) angiotensin-converting enzyme (ACE) inhibition activity; (**c**) iron-chelating activity, (**d**) oxygen radical absorption capacity (ORAC); (**e**) 2,2′-azinobis(3-ethylbenzothiazoline-6-sulfonic acid (ABTS) scavenging activity and (**f**) 2,2-diphenyl-1-picrylhydrazyl (DPPH) scavenging activity. Casein and oat proteins were digested *in vitro* and fed to the rodents (whole diet) and as pure proteins (protein isolate) to assess the impact of the food matrix on bioactive potency. Data are expressed as mean ± standard deviation (n = 3), and means without a common letter differ (*p* < 0.05) as analyzed by two-way ANOVA and Tukey’s test. For the DPPH assay, means without a common letter differ (*p* < 0.05) as analyzed by Student’s *t*-test.

As shown in Figure 9a, the *in vitro* gastrointestinal digestion of the whole feed led to a significantly higher (*p* < 0.05) degree of protein hydrolysis compared to the isolated protein for both oat and casein. For the ACE inhibition activity (Figure 9b), no significant differences (*p* > 0.05) were found between isolated oat proteins and casein, but a significantly lower activity (*p* < 0.05) was found for the oat-based diet compared to the casein-based diet.

The antioxidant activity was measured with a combination of *in vitro* assays targeting various mechanisms of action of antioxidants, including metal chelation, free radical scavenging through single-electron transfer (SET), and free radical scavenging through hydrogen atom transfer (HAT) (Figure 9). The results revealed that the oat protein isolate had significantly higher iron-chelating activity than casein, as demonstrated by the significantly lower EC_50_ (*p* < 0.05) (Figure 9c). No significant differences, however, were found for the oat- and casein-based diets. The results showed also no significant differences between casein and oat proteins in the ORAC assay for both the isolates and the diets (Figure 9d). In the ABTS assay, a slightly higher free radical scavenging activity was observed for the casein-based diet compared to the oat-based diet, but no significant differences were found between the isolates (Figure 9e). However, in the DPPH assay, oat proteins had a significantly higher antioxidant activity (lower EC_50_) than casein for both the isolates and the whole diets. The free radical scavenging activity of the casein-based diet was too low to determine the EC_50_ in the DPPH assay, whereas an EC_50_ was accurately determined for the oat-based feed.

**Figure 10:**
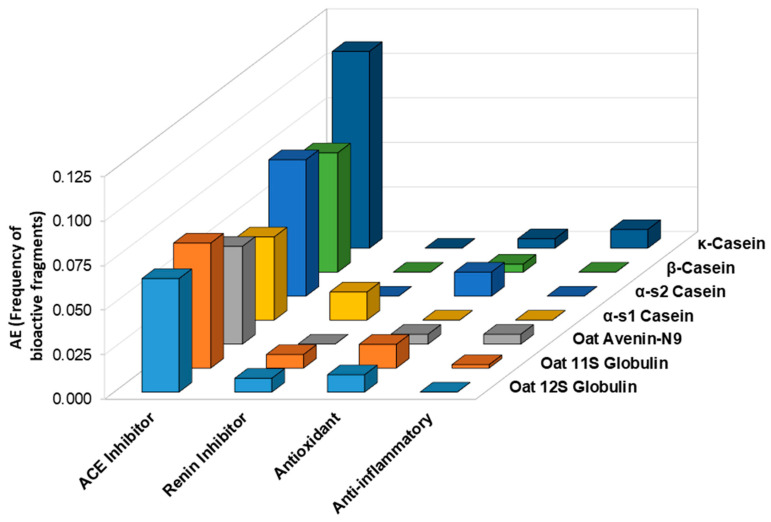
Predicted frequency of antioxidant, anti-inflammatory, and antihypertensive peptides after gastrointestinal digestion of casein and oat proteins according to the BIOPEP-UWM database.

An *in silico* analysis was further conducted with the BIOPEP database to predict the release of antihypertensive, antioxidant, and anti-inflammatory fragments after the gastrointestinal digestion of oat and casein proteins. As shown in Figure 10, a higher frequency of bioactive fragments with ACE inhibitory activity was found for caseins compared to oat storage proteins, particularly for κ-casein. Renin inhibitor fragments were found after the *in silico* gastrointestinal digestion of oat 11S and 12S globulins. In terms of antioxidant fragments, α-s2 casein had the highest frequency (A_E_ = 0.014), but oat 11S and 12S globulins were not far behind with A_E_ values of 0.013 and 0.010, respectively. Indeed, oat globulins had a higher frequency of antioxidant fragments than β-casein (A_E_ = 0.005), κ-casein (A_E_ = 0.005), and α-s1 casein (A_E_ = 0). Interestingly, for anti-inflammatory activity, both oat avenin and 11S globulin had fragments with predicted anti-inflammatory activity (A_E_ = 0.0055 and A_E_ = 0.0019, respectively). For the casein proteins, κ-casein had the highest frequency (A_E_ = 0.0105), but the other caseins had no fragments with predicted anti-inflammatory activity.

## 4. Discussion

We report for the first time that feeding an oat protein diet resulted in significant reductions in systolic and diastolic BPs in male SHRs in comparison to a regular diet containing casein in this protein-source-replacement study. Specifically, 16 weeks of feeding an oat protein diet was able to bring about mean decreases in systolic and diastolic BPs by approximately 29 and 25 mm Hg, respectively, in male SHRs. Our previous study showed that 15 weeks of treatment with oat beta-glucan reduced BP by approximately 20 mm Hg [23]. As previously reported, the systolic BP in male SHRs is around 182 mm Hg, which is known as hypertensive emergency that require urgent BP-lowering therapy to prevent organ damage [33]. This includes safely reducing BP immediately, limiting organ damage, and improving symptoms and clinical outcomes such as morbidity and mortality [33]. Even though our study was not aimed at treating hypertensive emergencies, in light of the current findings, it bears mentioning that managing chronic hypertension with interventions such as oat protein may help prevent the development of hypertensive emergencies. It should be noted that the OmniHeart randomized trial, which investigated the role of different macronutrient compositions on BP, demonstrated that a diet rich in protein had additional benefits including greater reductions in BP and improvements in lipid profiles [34]. Vegetarians who consume higher amounts of plant protein generally have a lower BP [35,36]. Similarly, oats, or extracts rich in oat beta-glucan, have also been effective in reducing the diastolic BP among individuals with obesity with high cholesterol. Arginine and tryptophan, especially the arginine in oat protein, are involved in nitric oxide production [36]. Moreover, oat-derived protein digests and peptides exhibit ACE and renin inhibitory activity [37,38,39]. Oat protein digest has also been reported to have a BP-lowering action in SHRs [39]. Similarly, plant-based proteins such as quinoa, wheat, and soy protein have been shown to improve BP in SHRs, suggesting the role of plant protein in lowering BP [40,41]. It would be worthwhile to explore the efficacy of protein in comparison to the antihypertensive mentioned above. Recently, alterations in the gut microbiota have been directly attributed to high BP [42]. For instance, gut bacterial fermentation metabolites, called short-chain fatty acids (SCFAs), are considered regulators of BP, and amino acids derived from protein are precursors of SCFAs. Of note, protein intake also correlates positively with overall microbial diversity. Precisely, oat protein significantly enhances the Bacteroidetes ratio (B/F ratio) and the abundance of *Muribaculaceae* while diminishing that of *Erysipelotrichaceae* in hypercholesterolemic hamsters [43]. On the other hand, the trimethylamine N-oxide (TMAO) derived from dietary choline and L-carnitine through gut microbiota is now recognized as a contributor to hypertension [44]. Recently, the Study with Appetizing Plantfood—Meat Eating Alternative Trial (SWAP-MEAT) showed that consuming plant-based alternative meat is inversely associated with TMAO levels [45]. In consideration of these possibilities, we propose that oat protein may also have a positive effect on microbial diversity, thereby promoting better BP control.

Hypertensive heart disease mainly manifests with signs of cardiac remodeling involving LV hypertrophy [46]. The administration of an oat protein diet was able to partially prevent hypertension-induced cardiac hypertrophy in SHRs at 20 weeks of age. Transthoracic echocardiography analysis showed that SHRs fed a regular diet had increased internal dimensions of the heart chamber, such as the left ventricle (LVID), which suggested that there was significant pathological cardiac dilation. Previous studies consistently showed that treating hypertension effectively can lead to improvements in left ventricular hypertrophy [47,48]. Overall, oat protein exhibits potential benefits in mitigating cardiac structural changes in the setting of severe hypertension.

Cardiac remodeling is also associated with cardiac dysfunction. In terms of cardiac systolic function, there was a significant reduction in the systolic functional parameters LVEF and FS in SHRs receiving a regular diet. This suggested that cardiac function deteriorated in the setting of uncontrolled hypertension and LV dilatation. SHRs fed the oat protein diet were found to have significantly higher LVEF and FS. This observation clearly indicated that the treatment effectively prevented systolic dysfunction by preserving the LVEF and FS. LVEF has prognostic significance; in heart failure with reduced LVEF, improving the LVEF is associated with significant clinical benefit [49]. Interestingly, our previous study showed that oat ingredients such as beta-glucan, avenanthramide C, or a combination of beta-glucan and avenanthramide C were unable to recover the LVEF in 20-week-old SHRs; nonetheless, beta-glucan treatment for 15 weeks in male SHRs had a significant antihypertensive effect [50]. Diastolic LV filling and LV ejection are invariably linked, and the pathophysiology of the diastolic dysfunction is therefore also linked. Diastolic dysfunction is caused by pathological changes in LV relaxation [48]. Inconsistent and incomplete relaxation of the LV causes decreased LV filling. Longer-than-normal IVRT seen on echocardiography indicates impaired relaxation of the LV diastolic phase. Patients with hypertension commonly exhibit an elongation of the IVRT, signifying diastolic dysfunction [51,52,53]. In this study, there was a prolongation of the IVRT in SHRs on the regular diet. Oat protein resulted in a reduction in the IVRT in male SHRs, suggesting that it was effective in preventing diastolic dysfunction by improving LV relaxation.

The genesis and evolution of high BP in SHRs is multifactorial, involving polygenetic characteristics that lead to renal dysfunction, vascular dysfunction, and neurohumoral dysregulation. These pathological irregularities are also invariably associated with oxidative stress, which in turn further exacerbates endothelial dysfunction and vascular damage, leading to end organ damage. Male SHRs fed a regular diet had significantly higher serum MDA (a marker of oxidative stress) in comparison to male WKY rats fed a regular diet at 20 weeks (Figure 7A). This observation further underscores the findings in the literature that oxidative stress tends to play a key role in the pathological changes in the setting of hypertension [54]. The serum MDA levels in male SHRs fed an oat protein diet were not significantly different between the groups. In this study, it was revealed that the oat protein diet was not associated with a significant lowering of the oxidative stress marker MDA, even though there was trend toward the lowering of MDA. Hence, the BP-lowering effect observed with oat protein may not be associated with preventing lipid peroxidation.

The levels of vasoconstrictor molecules, norepinephrine and Ang II, were comparable between the groups. Of note, a drift toward an increased level of Ang II was observed in the male SHRs on a regular diet. The exact opposite was observed: the Ang II levels in the oat-protein-diet-fed SHRs was closer to that in male WKY rats fed a regular diet. This observation may suggest that the initial neurohormonal upregulation was no longer present by 20 weeks of age in the SHRs in this study. A longer-term study may reveal if oat protein can be beneficial in further lowering the levels of Ang II. The serum levels of nitric oxide were significantly lower in male SHRs on the regular diet in comparison to WKY rats on the regular diet. The serum levels of nitric oxide were significantly higher in male SHRs on the oat protein diet than in SHRs on the regular diet. The dysregulation of nitric-oxide-mediated vascular homeostasis contributes to the development of hypertension via adversely affecting endothelial function [55]. Our finding suggests that oat protein is able to improve NO and thus NO-mediated vascular function, leading to an amelioration of hypertension in SHRs. Further studies are needed to identify whether oat protein increases the activity or level of eNOS and in turn results in higher levels of NO. NO is produced by an eNOS -mediated reaction [55]. eNOS is regulated by calcium/calmodulin via calcium calmodulin protein kinase II (CaMK II)-mediated phosphorylation (of eNOS) and by shear stress [56]. L-arginine is the substrate for eNOS, while molecular oxygen and nicotinamide-adenine-dinucleotide phosphate are co-substrates. Additionally, flavin adenine dinucleotide, flavin momonucleotide, and (6-R-)5,6,7,8-tetrahydro-L-biopterin (BH4) are cofactors for eNOS [57]. NO directly nitrosylates soluble guanylate cyclase (GC) in the vascular smooth muscle cells, catalyzing the formation of cyclic guanosine monophosphate (cGMP), which in turn phosphorylates target proteins, resulting in smooth muscle relaxation [58]. Given the fact that we observed a significant lowering of NO in the SHRs and a significant recovery of NO in the oat protein SHR group, it would be interesting to assess the status of the aforementioned mechanisms that lead to eNOS activation, such as eNOS phosphorylation by CaMKII as well as the levels of eNOS substrates, co-substrates, and cofactors in the vascular tissues of WKY rats and SHRs with and without oat protein. In this regard, it is pertinent to note that oat protein has a significant amount of L-arginine, the substrate for eNOS, and may have contributed to the beneficial effects of oat protein observed in the current study [12,57].

TNF-α is one of the most potent proinflammatory molecules: it induces inflammation by binding to its receptor on the cell, TNFR 1 [59]. TNF-α has also been thought to play a role in hypertension in male SHRs [60]. This binding leads to the activation of two pathways, the mitogen-activated kinase (MAPK) pathway and the canonical nuclear factor kB (NF-kB) pathway, resulting in the transcription of proinflammatory cytokines and chemokines, leading to inflammation [61]. TNF-α has been reported to decrease the expression of eNOS in endothelial cells [62,63]. In patients with hypertension, the levels of tumor necrosis factor-α have been shown to be elevated [64]. TNF-α has been linked to inflammatory kidney damage caused by hypertension [65]. TNF-α was significantly higher in male SHRs fed the regular diet. TNF-α was significantly lower in male SHRs fed an oat protein diet. This might suggest that oat protein effectively targets the TNF-α-mediated inflammation in SHRs. It should be noted that previous studies showed that TNF-α blockade with an inhibitor such as etanercept was not able to lower BP in SHRs, even though inflammation was reduced. This further underscores that approaches like oat protein offer a concomitant reduction in BP and inflammation via pleotropic effects [60]. Also, it would be interesting to study the mechanisms underlying the oat-protein-mediated reduction in TNF-α activity in SHRs fed oat protein observed in the current study by examining the binding of TNF-αto it is receptor TNFR1, and the activities of MAPK and NFkB in the vascular tissues of WKY rats and SHRs fed an oat protein or not.

The number of studies that have evaluated the association of AST and ALT with hypertension are few, and their results have been ambiguous. ALT and AST were significantly higher in male SHRs on a regular diet in comparison to male WKY rats on a regular diet at 20 weeks. ALT but not AST was significantly lower in male SHRs on the oat protein diet in comparison to male SHR rats on the regular diet at 20 weeks, suggesting that oat protein may partially improve liver function. Total, LDL, and HDL cholesterol were lower in male SHRs on an oat protein diet. However, the ratio of HDL to LDL was the highest in the SHRs on an oat protein diet, suggesting a lower risk of heart disease in these animals. These results are consistent with those of our previous study, which showed that oat protein significantly decreased serum total and low-density lipoprotein cholesterol levels and prevented increases in liver HMG-CoAR activity in high-fat-, high-sugar-fed Wistar rats [20]. Triglycerides were comparable between the groups, whereas glucose levels were lower in SHRs fed a regular diet, suggesting that oat protein did not have any effects on either of these parameters. Creatinine levels were lower in male SHRs on an oat protein diet, suggesting that oat protein may have positive effects on renal function in the setting of hypertension. Urea was also lower in male SHRs on an oat protein diet. The higher urea levels in SHRs fed a regular diet indicates a deterioration in renal function; therefore, their reduction in the SHRs on an oat protein diet suggests that oat protein may have positive effects on renal function in the setting of hypertension. In light of our findings, interestingly, it should be noted that an observational study in 5316 adults showed that each 20 g increase in plant protein intake correlated with a 16% decrease in chronic kidney disease, signifying that plant-based protein is able to protect renal function [66]. In addition, in a cross-sectional study stratified by estimated glomerular filtration rate (eGFR), it was found that for participants with an eGFR < 60 mL/min/1.73 m^2^, every 33% increase in the ratio of plant protein to total protein was associated with a 19% lower risk of mortality [67]. In addition, as an isocaloric protein substitution study, the present study yields crucial insights into the impact of oat protein on hypertension, without confounding factors such as variations in caloric intake. Moreover, oat protein may offer protective effects against the deterioration of renal function observed in conditions like hypertension. It further highlights a promising approach involving plant-based dietary modifications to mitigate cardiovascular disease in the setting of hypertension. Importantly, this strategy may also facilitate reducing the dosage of antihypertensive medications, as the aggressive BP-lowering mandated for cardiovascular protection through drug therapy is prone to producing major side effects such as renal dysfunction. Further understanding the role and mechanisms of action of an oat protein diet on the microbiota, the tissue-level oxidative stress, inflammation, and endothelial nitric oxide synthase will extend our knowledge and address pertinent questions that need to be answered to achieve its utilization.

The gastrointestinal breakdown of dietary protein produces peptides with antihypertensive effects, including ACE inhibitor peptides, which could explain the BP-lowering effect of oat protein when fed to SHRs [68,69]. To investigate this, the ACE inhibitory activity of oat- and casein-protein-based feeds and isolates after *in vitro* gastrointestinal digestion was measured. Surprisingly, oat protein did not show higher ACE inhibition activity compared to casein, and oat-based feed had an even lower ACE inhibitory activity compared to the casein-based feed. The results of the predictive *in silico* bioactivity analysis revealed the same tendency.

Therefore, the stronger BP-lowering effect observed *in vivo* in rats fed an oat-based diet compared to the casein-based diet could have resulted from synergistic effects on other components of the RAAS, such as renin and/or ACE 2, contributing to the better BP-lowering effect of the oat-based compared to the casein-based diet [70]. The *in silico* activity revealed the presence of renin inhibitory fragments after the digestion of oat proteins, further supporting this hypothesis. Moreover, oat-derived ACE inhibitory peptides could possibly have better bioavailability compared to the casein-derived ones, explaining the disparity between the *in vivo* and *in vitro* results. Nonetheless, oat proteins after gastrointestinal digestion still have an interesting inhibitory effect against ACE, which is likely to contribute to the overall blood-pressure-lowering effect observed *in vivo*.

The release of peptides with antioxidant activity after gastrointestinal digestion can also contribute to the cardioprotective effects and BP-lowering effects of oat proteins observed *in vivo*. Indeed, increased oxidative stress has been linked to the development of hypertension [71] by causing endothelial and renal damage, vascular dysfunction, and cardiovascular fibrosis [54]. Hence, the antioxidant activity of the 3 kDa permeates of oat and casein protein gastrointestinal digestate was assessed with a combination of *in vitro* antioxidant assays. The results revealed that oat proteins after gastrointestinal digestion had higher antioxidant activity than casein when assessed by the DPPH and the iron-chelating assays, suggesting that the antioxidant activity of oat peptides generated after gastrointestinal digestion may have contributed to the observed BP-lowering effect *in vivo*.

For both the ACE inhibition and the antioxidant activities, there were significant differences between the isolates and the whole feed, revealing an important effect of the matrix. Overall, the bioactive potency of the protein isolates was higher compared to the whole feed after *in vitro* gastrointestinal digestion, except for the iron-chelating activity. These differences in bioactive potency can be explained by their digestibility, as demonstrated by the protein DH data. Interestingly, the whole feed was more readily digestible than the protein isolates. This could be attributed to the higher degree of processing (i.e., extrusion) performed during feed preparation [72], resulting in further protein denaturation, thereby facilitating protein hydrolysis by the digestive enzymes. The observed differences in the hydrolysis between the different matrices (protein isolate versus whole feed) could have resulted in the differences in the digestates’ peptides profiles and bioactivities. Indeed, peptide bioactivity is highly dependent on peptide length, amino acid composition, and sequences [73]. Moreover, the oat protein isolate used in this study was not 100% pure and still contained up to 10% of other oat components such as starch and polyphenols. These constituents, exclusive to plant-based matrices, are known to react with proteins under the high temperature and/or high pressure applied during food processing [74,75], resulting in various reactions such as glycation, dehydration, and oxidation, which can affect bioactive potency [76].

In summary, an oat protein diet had no significant impact on angiotensin II *in vivo* and did not have a greater impact on ACE inhibition *in vitro*, suggesting that ACE inhibition may not be the principal target leading to lowering the BP in SHRs. Based on the *in vivo*, *in vitro*, and *in silico* results, the antioxidant and anti-inflammatory effects of oat proteins after *in vitro* gastrointestinal digestion would be a promising mechanistical target for further investigation.

## 5. Conclusions

In conclusion, this study strongly suggests that incorporating oat protein into the diet may have beneficial effects on BP and hypertension-induced cardiac remodeling and dysfunction partly via improving nitric oxide and reducing inflammation in SHRs. Additionally, more studies are needed to fully understand its mechanism of action and its potential long-term clinical efficacy as an antihypertensive treatment strategy.

## Data Availability

Available upon reasonable request.
